# Shared and unique responses of plants to multiple individual stresses and stress combinations: physiological and molecular mechanisms

**DOI:** 10.3389/fpls.2015.00723

**Published:** 2015-09-16

**Authors:** Prachi Pandey, Venkategowda Ramegowda, Muthappa Senthil-Kumar

**Affiliations:** ^1^National Institute of Plant Genome ResearchNew Delhi, India; ^2^Department of Crop Physiology, University of Agricultural SciencesBangalore, India

**Keywords:** tailored response, unique adaptation mechanisms, drought, heat, pathogen infection, concurrent stress

## Abstract

In field conditions, plants are often simultaneously exposed to multiple biotic and abiotic stresses resulting in substantial yield loss. Plants have evolved various physiological and molecular adaptations to protect themselves under stress combinations. Emerging evidences suggest that plant responses to a combination of stresses are unique from individual stress responses. In addition, plants exhibit shared responses which are common to individual stresses and stress combination. In this review, we provide an update on the current understanding of both unique and shared responses. Specific focus of this review is on heat–drought stress as a major abiotic stress combination and, drought–pathogen and heat–pathogen as examples of abiotic–biotic stress combinations. We also comprehend the current understanding of molecular mechanisms of cross talk in relation to shared and unique molecular responses for plant survival under stress combinations. Thus, the knowledge of shared responses of plants from individual stress studies and stress combinations can be utilized to develop varieties with broad spectrum stress tolerance.

## Introduction

Under field conditions, plants are concurrently exposed to a number of abiotic and biotic stresses. Stress combinations instead of individual stresses have been recognized as realistic threats faced by plants (Rizhsky et al., 2004; Mittler, 2006; Kissoudis et al., 2014; Suzuki et al., 2014; Mahalingam, 2015; Ramegowda and Senthil-kumar, 2015). Therefore, for development of plants with better adaptation under field conditions, focus should now be diverted toward understanding plant responses under combined stress conditions. Simultaneous occurrence of different biotic and abiotic stresses results in deployment of stress-adaptation strategies which are different and sometimes contrasting to those seen under individual stresses. For example, under combined drought and heat stress, *Arabidopsis thaliana* plants accumulate sucrose instead of proline (Rizhsky et al., 2004). The enhanced transpiration to cool leaf surface during heat stress aggravate the effects of concurrent drought and salinity because increased transpiration rate leads to more water loss and increased uptake of salts (Rizhsky et al., 2002; Mittler, 2006). Concurrent occurrence of an abiotic stress with a biotic stress either aggravates or inhibits the effect of latter leading to either enhanced or reduced susceptibility to pathogens (Audenaert et al., 2002; Mohr and Cahill, 2003; Ton and Mauch-Mani, 2004; Melotto et al., 2006; Adie et al., 2007; Asselbergh et al., 2008; Ramegowda and Senthil-kumar, 2015). Thus, abiotic stresses can strongly modulate plants tolerance or susceptibility toward pathogen by different mechanisms which include trade-off between biotic and abiotic stress responses, and lead to modification in plant–pathogen interactions. This presents the need to study physiological and molecular responses of plants under abiotic and biotic stress combinations in order to understand plants tolerance against stress combinations.

The abiotic and biotic stress signaling networks of plants consist of several interacting pathways (Knight and Knight, 2001; Smekalova et al., 2014). Different abiotic and biotic stress conditions lead to some common physiological and molecular processes in plants apart from the unique responses. Plant adaptation strategy to a combination of two stresses consists of both “shared” and “unique” response. Shared responses refer to the molecular and physiological responses which are common to the two different stresses and unique responses are the ones which are specific to the individual stresses or the stress combinations (Rizhsky et al., 2002, 2004; Atkinson et al., 2013; Narsai et al., 2013; Prasch and Sonnewald, 2013; Sewelam et al., 2014; Supplementary Figure 1). Shared mechanisms constitute a considerable portion of plants response to both individual and combined stresses. These mechanisms include production and detoxification of reactive oxygen species (ROS), calcium-, phytohormone-, and MAPK-signaling pathway (Xiong and Yang, 2003; Li et al., 2008; Atkinson and Urwin, 2012; Suzuki et al., 2012, 2014; Rejeb et al., 2014). The shared responses are general physiological adaptation of plants and can guard them against multiple individual stresses. Some unique adaptation strategies tailored for stress combinations have been identified in the recent reports (Mittler, 2006; Atkinson et al., 2013; Choi et al., 2013; Prasch and Sonnewald, 2013). For example, combination of heat stress and virus infection led to up-regulation of cytosolic invertases instead of cell wall bound invertases (Prasch and Sonnewald, 2013).

Among different stress combinations that occur in field conditions, heat and drought stress and their interaction with pathogens are the most studied (Rizhsky et al., 2002, 2004; Mittler, 2006; Prasad et al., 2011; Bostock et al., 2014; Rejeb et al., 2014; Suzuki et al., 2014; Pandey et al., 2015; Ramegowda and Senthil-kumar, 2015). Therefore, taking these stresses as representatives of abiotic–abiotic and abiotic–biotic stress combinations, we enumerate the unique and shared responses exhibited by plants under drought–heat, drought–pathogen, and heat–pathogen combinations. We also provide a comparison of the overlap between cross talk of signaling pathways identified from multiple individual stress studies and the shared responses identified from combined stress transcriptome studies. Such overlapping responses can be a source of potential stress tolerance traits that can be engineered into crops to confer multiple stress resistance into plants.

## Delineation of shared and unique responses in abiotic stress combinations

**Figure 1:**
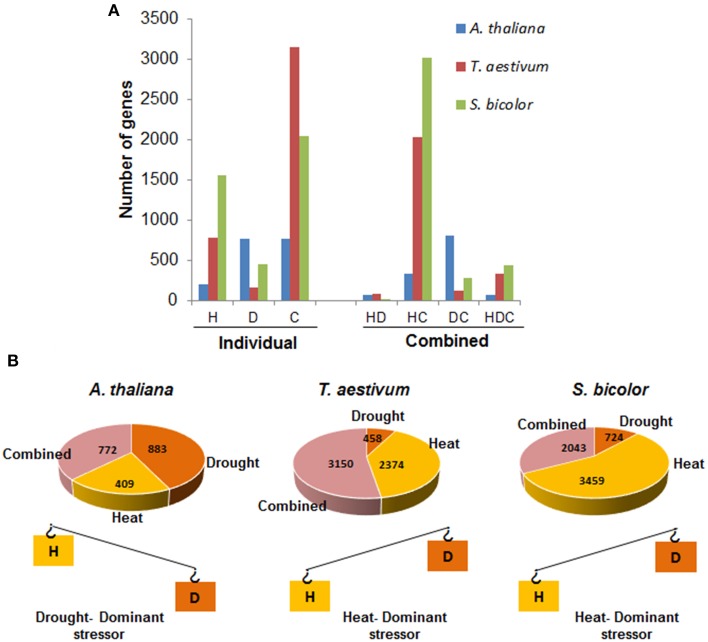
**Representation of unique and shared responses and the “dominant stressor” concept in *A. thaliana*, *T. aestivum*, and *S. bicolor* under combined heat and drought stress**. **(A)** H, D, and C denote the number of genes modulated (refer to both up- and down-regulated) exclusively under heat, drought, and combined heat and drought stress, respectively. HD, HC, DC, and HDC represent the commonly regulated genes under heat and drought stress, heat and combined, drought and combined stresses, and all the three stresses, respectively. The figure is a graphical representation of the data (number of genes modulated under the different stress condition) provided in three independent cDNA array studies in *A. thaliana, T. aestivum*, and *S. bicolor* by Rizhsky et al. (2004), Aprile et al. (2013), and Johnson et al. (2014), respectively. **(B)** Representation of the “dominant stressor concept” under combined stress. The rectangles represent heat and drought stress. In a given stress combination, two stresses involved differ in severity of impact on plants. The severity of the two stresses is represented by “see saw.” In case of *A. thaliana*, the molecular responses seen are drought specific with a maximum overlap between genes modulated under drought and combined stress. In *T. aestivum*, the number of heat stress specific genes outweighs the number of drought specific genes. However, the number of combined stress-specific genes is far greater than the individual stress specific genes and molecular response to the combined stress conditions is mostly unique in this plant. In case of *S. bicolor*, the number of genes specific to heat stress outweighs the number of drought stress specific genes. The genes commonly modulated (both up- and down-regulated) under heat and combined stress forms the maximum share in the combined stress response. H, heat; D, drought; C, combined stress. The pie chart represents the molecular response of plants to the combined stress and the area denotes the number of genes modulated under each category.

**Figure 2:**
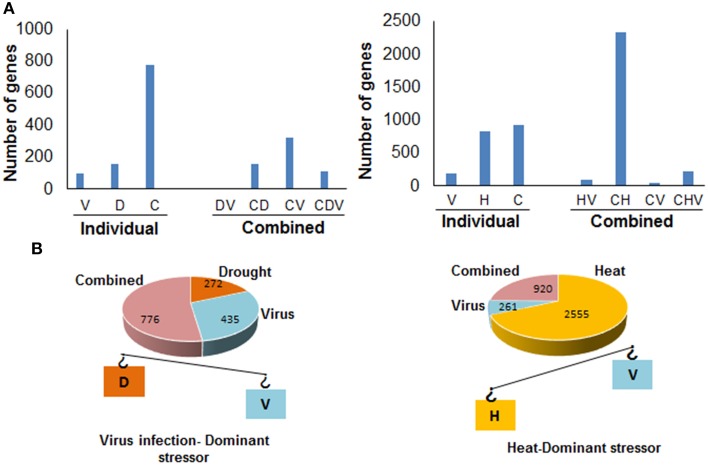
**Unique and shared responses and the “dominant stressor” concept in *A. thaliana* under combined heat, virus, and drought stress**. **(A)** Representation of unique and shared responses of *A. thaliana* under drought, virus infection, and their combination(left) and heat, virus, and their combination (right). The bar diagram (left) represents the number of genes modulated exclusively under virus (V), drought (D), combined heat and drought stress (C) as well as the number of commonly regulated genes under virus infection and drought stress (DV), drought and combined (DC), virus and combined stresses (VC), and all the three stresses (CDV). The bar diagram at the right represents the number of unique genes modulated exclusively under virus (V), heat (H), combined heat and virus stress (C) as well as the number of genes commonly regulated under heat and virus infection (HV), heat and combined (CH), virus and combined stresses (CV), and all the three stresses (CHV). **(B)** The figure represents the dominant stressor concept. Drought and virus stress are represented by orange and blue rectangles. In this case, virus infection has more effect on the gene expression of *A. thaliana* plants. The number of genes unique to combined stress is far greater than that of individual stress genes and molecular response to the combined stress conditions is mostly unique. Heat and virus stress are represented by yellow and blue rectangles. In this case, heat stress has more effect on the gene expression. The number of heat and combined stress genes are nearly same and molecular response to the combined stress conditions mostly consists of genes commonly modulated under heat and combined stress. The figure is a graphical representation of the data provided in microarray study by Prasch and Sonnewald (2013). H, heat; D, drought; C, combined stress.

When two stresses occur concurrently, the adaptation strategy of plants to stress combination is governed by the interaction of two stresses which is conceived by plants as a new state of stress (Mittler, 2006). Thus, adaptation strategies of plants to combined stress may be different from that of two individual stresses. The overall effect of stress combination on plants depends largely on the age of plant, the inherent stress-resistant or susceptible nature of plant and severity of two stresses involved. Plant responses to stress combination are majorly determined by the more severe stress (dominant stressor, Figures 1B, 2B) such that the physiological and molecular processes of plants subjected to combined stress resemble with those observed under more severe individual stress.

The shared responses under combined stresses constitute the generic morpho-physiological and molecular events evoked by both stresses constituting stress combination (Supplementary Table 1). For example, drought, salinity, and chilling induce osmotic effect on plants resulting in induction of common physiological processes, one of which is accumulation of osmoprotectants (Chinnusamy et al., 2004). The other stress induced response shared by almost all abiotic stress conditions is the production of ROS. Heat and salt stress are known to commonly affect the transport and compartmentation of ions in plants (Munns, 2002). Drought and salinity stress evoke the generic response of creating a physiological water deficit in plants. Additionally, both stresses cause decreased CO_2_ diffusion into chloroplast due to reduced stomatal opening leading to reduced carbon metabolism.

In addition, some physiological traits are unique to individual drought, heat, salinity and chilling stress (Supplementary Table 1). For example, in salinity and chilling stress, ion-compartmentation and regulation of ice nuclei formation, respectively, are the unique responses (Chinnusamy et al., 2004). Salinity stress specifically disturbs the ion homeostasis by increased Na^+^ and reduced K^+^ uptake. Similarly, heat stress causes changes in membrane fluidity, affecting ion transporters, and pumps thereby disrupting ion transport (Plieth et al., 1999; Conde and Chaves, 2011).

The interaction between two stresses can either be additive or antagonistic to each other. The combination of drought and salinity is an example of additive interaction between the two stress conditions (Supplementary Table 1). Concurrent drought and salinity affected the growth of *Hordeum spontaneum* (wild variety of barley) more severely than the individual stresses (Ahmed et al., 2013). The individual and combined drought and salt stress led to drastic inhibition of net photosynthetic rate, stomatal conductance, and enhanced oxidative damage. Combined stress also resulted in enhanced reduction of chlorophyll b as compared to that observed under individual stresses. Overall, interaction between the two stress conditions was found to be additive for almost all the physiological parameters resulting in enhanced damage to plants under stress combination. Some differences in responses to individual and combined stress have also been noticed. For example, combined drought and salt stress led to enhanced Na^+^ accumulation in roots as compared to leaves and stems whereas under salinity stress Na^+^ preferably accumulated in shoots (Ahmed et al., 2013).

The combined stress mitigation strategy of plants also constitutes some unique morpho-physiological processes which makes the overall response of plants to stress combination different from that seen under individual stresses (Supplementary Table 1). For example, although both heat and salt stress are damaging to plants, concurrent salinity with heat stress enhanced salt tolerance of *Solanum lycopersicum* (Rivero et al., 2014). The combined heat and salt stress led to Na^+^ accumulation in roots rather than in leaves and shoots. Thus, heat stress resulted in salinity tolerance by inhibiting uptake of Na^+^ ions and by directing the accumulation of Na^+^ to roots rather than shoots (Rivero et al., 2014). *S. lycopersicum* plants treated with combined heat and salinity stress accumulated the osmoprotectants glycine betaine and trehalose in large amounts instead of proline which is a predominant osmoprotectant accumulated in plants challenged with salinity stress only. Under individual salt stress, activity of the enzyme 1-pyrroline-5-carboxylate synthase (P5CS) increased indicating the synthesis of proline from glutamate. However, under combined stress, a decrease in the activity of P5CS and increase in the activity of ornithine aminotransferase (OAT) was observed suggesting that under the combined stress, proline synthesis occurred from ornithine through ornithine aminotransferase (OAT). The occurrence of proline synthesis through OAT has been observed in plants under some conditions (Krell et al., 2007, reviewed in Verslues and Sharma, 2010). Taken together, enhanced accumulation of glycine, betaine, and trehalose improved tolerance of plants exposed to combined stress (Rivero et al., 2014).

To further explicate the distinct and shared mechanisms of plants response to individual and combined abiotic stress conditions, we selected drought and heat stress combination as an example and hereby describe their effects on physiological and molecular processes. The enhanced damage incurred by heat and drought stress combination as compared to individual stresses is due to the fact that heat and drought share a number of physiological traits and the overall effect of the two stresses on plants is additive and leads to aggravated stress effects. However, the two stress conditions also evoke unique responses as outlined in sections below. In a study conducted by Rollins et al. (2013) on two genotypes (Arta and Keel) of *Hordeum vulgare*, drought stress was found to have stronger effect on traits like plant height, biomass and spike number whereas reproductive traits like number of aborted spikes and kernel weight were more affected by heat stress (Rollins et al., 2013).

## Morpho-physiological responses of plants to drought and heat stress combination

The mechanism of adaptation to drought and heat stress varies considerably which results in unique morphological responses under these stresses. Plants adapt to drought stress by minimizing water loss and increasing water uptake. This is achieved by reducing leaf number, area, and increasing root growth by plants. On the other hand, long term adaptive strategies for heat tolerance encompass decreasing the leaf canopy temperature through increased transpiration by increasing leaf number and area. Drought and heat stress have contrasting effect on some morphological processes. For example, leaf expansion, leaf number, and size were reduced due to drought stress (Alves and Setter, 2004) while heat stress led to increase in leaf number and leaf elongation (Bos et al., 2000; Prasad et al., 2006). Heat stress was shown to decrease number, length, and diameter of roots but moderate drought stress increased root growth which is required for water uptake from deeper layers of soil (Prasad et al., 2008). Drought stress reduced the leaf area (Poorter et al., 2009) whereas heat stress led to production of thinner leaves with higher specific leaf area (Luomala et al., 2005; Poorter et al., 2009). On the other hand, biomass allocation to roots increased in response to drought while heat stress enhanced reproductive allocation. During combined stress, some of the responses were shared with drought and some with heat stress. For example, leaf size was found to increase, leaf number was decreased, and biomass allocation was seen to occur preferably in roots and reproductive parts under combined stress in *A. thaliana* (Vile et al., 2012).

Unlike the contrasting effect of drought and heat stresses on vegetative growth of plants, drought, heat and their combination had similar effects on the reproductive development of plants. These stresses have been shown to delay flowering, reduce grain weight and yield of *Triticum aestivum* (Savin and Nicolas, 1996; Prasad et al., 2006; Pradhan et al., 2012). The combined stress conditions were found to be more detrimental than the individual stresses in reducing yield of *H. vulgare* (Rollins et al., 2013). Drought and drought–heat combination reduced the spike number, which was not affected by heat stress. Similarly heat and combined stress increased the number of aborted kernels (Rollins et al., 2013). However, drought stress did not cause any change in size and nutrient accumulation in plant endosperm while combined heat and drought stressed plants produced enlarged endosperm with higher accumulation of starch and protein (Szucs et al., 2010).

Heat and drought stress differentially affect stomatal characteristics. Under combined heat and drought stress, stomata remained closed leading to increased leaf temperature of *Nicotiana tabacum* plants (Rizhsky et al., 2002). Plants under combined stress minimize leaf temperature in a unique way. Vile et al. (2012) reported that *A. thaliana* plants exposed to combined heat and drought stress adapt to heat stress by adjusting leaf orientation through increasing their leaf insertion angle. *A. thaliana* plants exposed to individual and combined heat and drought stresses showed increased stomatal density in response to drought stress which was reduced in response to heat stress. Under combined stress, however, stomatal density decreased (Vile et al., 2012). This suggests that in case of a stress combination constituting of two stresses differing in their severity, plant's physiological processes are apparently determined by the more severe stress. The combined heat and drought stress led to higher leaf temperature in two genotypes of *T. aestivum*, Ofanto and Cappelli which differ in water use efficiency (WUE). Cappelli is characterized by higher WUE and lower stomatal conductance compared to Ofanto. The combined stress led to a higher leaf temperature in Cappelli as compared to Ofanto (Aprile et al., 2013). This indicates that the effect of combined stress also varies among the genotypes of a particular plant species.

The combined heat and drought stress have been shown to affect a number of physiological processes more severely than the individual stresses. Rizhsky et al. (2002) reported that *N. tabacum* plants exposed to simultaneous heat and drought stress led to greater suppression of photosynthetic activity as compared to individual stresses. Similarly, as compared to individual stresses, combined heat and drought stress lead to enhanced lipid peroxidation in *Lotus japonicus* (Sainz et al., 2010) and severe abnormalities in the ultra-structure of chloroplasts and mitochondria in *T. aestivum* (Szucs et al., 2010; Grigorova et al., 2012). The combined stress also led to greater reduction in photosynthetic activity and enhanced production of ROS in *Populus yunnanensis* (Li et al., 2014) and greater diminution in root viability and photochemical efficiency of photosystem II (PS-II) in *Festuca arundinacea* (Jiang and Huang, 2001). The reduction in photosynthetic activity is a response shared between the individual heat and drought stresses. However, photosynthesis is less affected by heat stress and only high temperatures (>40°C) are known to be detrimental. Heat stress mediated reduction in photosynthesis mainly occurs due to enhanced photorespiration (Prasad et al., 2008), reduced RuBisCO activity (Salvucci and Crafts-Brandner, 2004), and reduced PS-II activity (Yang et al., 2007). Heat stress did not reduce photosynthetic activity of tobacco plants, but drought stress and combined heat and drought stress led to more than 80% reduction in photosynthetic activity (Rizhsky et al., 2002). The RuBisCO activity in *Cicer arietinum* leaves was increased with heat stress and decreased with drought stress and combined stress (Awasthi et al., 2014). Similarly, Sainz et al. (2010) reported significant disruption in PSII function when *L. japonicas* plants were subjected to combined heat and drought stress. Jiang and Huang (2001) compared the response of *F. arundinacea* and *Lolium perenne* to combined heat and drought stress and observed that stress combination led to enhanced reduction in photochemical efficiency of PS-II, as compared to individual stresses.

The modulation of mitochondrial respiration is also a shared response under drought and heat stress (Prasad et al., 2008). The rate of dark respiration increased with increasing temperatures whereas drought stress reduced plant respiration rates (Bryla et al., 2001). Similar observations were made by Rizhsky et al. (2002) who found that drought stress led to suppression of respiration but heat and combined drought and heat stress led to enhancement of respiration in *N. tabacum* leaves.

## Molecular response of plants to heat and drought stress combination

The transcriptomic analysis of combined heat and drought stressed *A. thaliana, N. tabacum, H. vulgare*, and *T. aestivum* by different groups have revealed a combination of shared and unique transcriptomic changes (Rizhsky et al., 2002, 2004; Rampino et al., 2012; Johnson et al., 2014). However, the transcriptomic changes are dependent on the plant type, duration and severity of stresses. In *A. thaliana* plants subjected to combined drought and heat stress (Supplementary Table 3), the molecular response under combined stress was dominated by drought specific transcriptomic changes and consisted of 208, 765, and 772 genes specifically modulated (refer to both up- and down-regulated) under heat, drought and combined stress, respectively. Furthermore, 77, 806, and 332 genes were commonly regulated under drought and heat, drought and combined stress and heat and combined stress, respectively (Rizhsky et al., 2004). In case of *T. aestivum* (var. Ofanto) plants heat stress response was found to be the most dominating (Supplementary Table 3). The combined stress led to modulation of 5645 transcripts out of which, 2037 and 121 were common with heat and drought stress, respectively, and 3150 transcripts were unique to combined stress. The transcripts modulated specifically under heat and drought stress totaled 159 and 779, respectively, with 90 transcripts commonly regulated under heat and drought stress response (Aprile et al., 2013; Figure 1). Rampino et al. (2012) studied gene expression profile of *T. aestivum* plants by cDNA amplified fragment length polymorphism (cDNA-AFLP). The study revealed that 380 genes were modulated in all the three stress conditions. Out of 242 up-regulated genes, 44, 15, and 90 genes were specifically induced by heat, drought and combined stress, respectively. While 18 genes were commonly up-regulated in heat and drought stress, 51 and 24 up-regulated genes were common among individual heat, drought and the combined heat and drought stress, respectively. Therefore, in case of *T. aestivum*, the response under combined stress constituted more of unique than shared response. Similarly, transcriptomic analysis of individual and combined stressed *Sorghum bicolor* plants (Supplementary Table 3) using DNA microarray revealed that 1554, 448, and 2043 genes were specifically modulated under heat, drought and combined stress whereas 18, 3021, and 286 of genes were found to be common under heat and drought, heat and combined stress and drought and combined stress, respectively (Figure 1). A total of 438 genes were commonly regulated under individual drought, heat and combined drought and heat stress conditions (Johnson et al., 2014). Thus, it is evident that the number of heat stress specific genes outweighs the number of drought specific genes in *T. aestivum* and *S. bicolor*. This may be due to the severe nature of the heat treatment. In case of *A. thaliana*, the molecular responses seen are drought specific. Moreover, in all the three plants, the number of combined stress-specific genes is more than the individual stress-specific genes showing thereby that the molecular response of these plants to the combined stress conditions is mostly unique. Plants have to maintain a balance between energy and resource allocation toward growth and stress adaptation. Thus, when simultaneously exposed to multiple stress conditions, they respond to the more damaging stress condition. This is evident from the gene expression studies which show that the molecular responses are more tuned toward heat stress in the above mentioned instances of *T. aestivum* and *S. bicolor* (Figure 1B).

The shared response under combined drought and heat stress constituted the induction of heat shock proteins (HSPs), ROS detoxification enzymes, and enzymes involved in photosynthesis and glycolysis (Rizhsky et al., 2002, 2004; Rampino et al., 2012; Johnson et al., 2014). Rizhsky et al. (2002) reported induction of genes encoding small HSPs (sHSPs), HSP70, HSP90, and HSP100 under individual as well as combined stress in *N. tabacum*. Apart from HSPs, the other genes constituting shared response under individual and combined stress include late embryogenesis 7 (*LEA7*) genes, dehydrin, photosynthesis related genes, and genes encoding enzymes involved in pentose pathway and anthocyanin biosynthesis (Rizhsky et al., 2004). Functional classification of genes commonly regulated under individual and combined drought and heat stress response in *A. thaliana* revealed that the largest class of commonly regulated genes was constituted by those involved in different metabolic processes (Supplementary Figure 2A). Chaperones formed the largest class of proteins commonly regulated under heat and combined stress. Transferases, oxidoreductases, and hydrolases encoding genes comprised the largest class of commonly regulated genes between drought stress and combined stress response (Supplementary Figure 2B; Rizhsky et al., 2004).

Although combined and individual stress response of plants constituted a number of commonly regulated genes, differences were observed in their expression levels in individual and combined stress conditions i.e., the expression was tailored to combined stress condition. For example, when compared to individual drought and heat stressed plants, combined stressed plants exhibited higher induction of HSP coding genes (Rizhsky et al., 2002). Differences were also seen in the type of ROS detoxification genes induced under the three stress conditions reflecting stress dependent ROS-detoxification mechanisms. For example, heat stress induced cytosolic ascorbate peroxidase (*APX*) and thioredoxin peroxidase (*TPX*). Drought stress led to the induction of catalase (*CAT*) and glutathione peroxidase (*GPX*). However, under combined stress, genes encoding alternative oxidase (AOX), GPX, glutathione reductase (GR), copper–zinc superoxide dismutase (CuZnSOD), and glutathione S transferase (GST) were found to be specifically induced.

Some unique genes were also found in the individual and combined stress conditions. For example, Sb02g038425 [homologous to resistance to *Pseudomonas syringae* pv. maculicola 1 (RPM1) protein] was found to be up-regulated exclusively under heat stress. Sb01g021320 (homologous to LEA D34 protein) and Sb05g017950 (*H. vulgare* aleurone 22 [HVA22] like protein) were found to be up-regulated exclusively under drought and combined stress (Johnson et al., 2014). Similarly, combined drought and heat stress led to the induction of various stress related genes which were not induced under individual stresses. These included genes encoding pathogenesis related (PR) and phenylalanine ammonia lyase (PAL) proteins. Induction of transcript encoding WRKY transcription factors and ethylene response transcriptional co-activator (ERTCA) were also unique to combined stress (Rizhsky et al., 2002). Other genes specifically elevated under combined stress are receptor-like kinases, protein kinases (*MAP3K*), small GTP-binding proteins, MYB transcription factors, transporters, aquaporin membrane intrinsic protein (*MIP*, Rizhsky et al., 2004), and genes encoding transcription factor WRKY8, calcium transporter ATPase9, heat shock protein cognate 70, and a disease resistance related protein (Rampino et al., 2012). Another unique response seen under combined stress was the down-regulation of gene encoding glycolate oxidase, which was otherwise induced under drought stress (Rizhsky et al., 2002). The metabolic overview map generated through MapMan (Supplementary Figure 3) revealed the induction of genes involved in carbohydrate and lipid metabolism under combined drought and heat stress in *A. thaliana* plants (Rizhsky et al., 2004).

## Plant responses to combined biotic and abiotic stresses

Occurrence of abiotic stresses such as drought, heat, cold, salinity, ozone, ultraviolet (UV) radiation, and nutrient stress dramatically alters the response of plants to biotic stresses. Similarly, interaction of plants with pathogens affects their responses to abiotic stresses. The outcomes of these interactions can either provide resistance or susceptibility toward any of the two stresses depending on the plant species, pathogen and stress intensity.

Abiotic stresses generally reduce some of obligate or biotrophic pathogen infection and severity of diseases (Schoeneweiss, 1975). For example, in *L. esculentum*, drought stress reduced infection of necrotrophic fungus *Botrytis cinerea* (causal agent of gray mold in tomato) by 50% and suppressed the biotrophic fungus *Oidium neolycopersici* (causal agent of powdery mildew in tomato) infection with concomitant two-fold increase in ABA compared to well-watered infected plants (Achuo et al., 2006). Conversely, hemibiotrophic pathogens can cause severe disease during drought stress even from low level of inoculum. For example, in *Carthamus tinctorius* drought stress increased the root rot caused by *Phytophthora cryptogea* (causal agent of root rot in safflower; Duniway, 1977). Long-term abiotic stress weakens plant defenses and causes enhanced susceptibility to pathogens (Amtmann et al., 2008; Goel et al., 2008; Mittler and Blumwald, 2010). This can also happen with increased colonization of pathogens in presence of abiotic stresses. For example, salinity increased colonization of roots by *Phytophthora cryptogea* in *Chrysanthemum morifolium* resulting in increased susceptibility of plants to the root rot (MacDonald, 1982). These evidences suggest that the outcome of combined abiotic stress and pathogen interactions may lead to increased severity of disease in the host plant.

Abiotic stresses can enhance disease resistance of plants through primed physiological adaptations (Kuwabara and Imai, 2009). For example, salicylic acid (SA) biosynthesis under cold temperatures with a corresponding induction of PR proteins minimized the impact of pathogens (Kim et al., 2013). Similarly, pathogen infection can also bring physiological adaptations in plants resulting in enhanced tolerance of plants to abiotic stresses. For example, in *A. thaliana, P. syringae* infection caused stomatal closure and prevented pathogen entry which resulted in reduced water loss from the infected plant, hence increasing the tolerance of plants to drought stress (Goel et al., 2008; Beattie, 2011). Similarly, infection of *A. thaliana* plants with soil borne fungal pathogen *Verticillium longisporum* (causal agent of wilt in thale cress) resulted in increased plant tolerance to drought stress due to *de novo* xylem formation resulting in enhanced water flow (Reusche et al., 2012). These evidences suggest that physiological adaptations caused by the prior stress can enhance tolerance of plants to the subsequent stress when plants are challenged with combination of biotic and abiotic stresses.

Presence of abiotic stress can arrest the infection ability of some pathogens. For example, salt-induced osmotic stress increased tolerance of *H. vulgare* plants to *Blumeria graminis* (causal agent of powdery mildew in barley) in a concentration dependent manner (Wiese et al., 2004). During this osmotic stress, papilla-mediated resistance resulted in callose deposition and this prevented fungal growth and infection. Similarly, presence of abiotic stress can increase the infection ability of some of the pathogens causing severe disease. For example, chilling increases susceptibility of *Gossypium* spp. to *Alternaria alternate* (causal agent of leaf spot in cotton), increasing leaf senescence and resulting in premature defoliation (Zhao et al., 2012). In *Oryza sativa*, low temperatures decreased resistance of plants to blast pathogen *Magnaporthe oryzae* (causal agent of blast in rice; Koga et al., 2004). Thus, the concurrent abiotic stress may directly modulate the plant-pathogen interactions leading either to enhanced or reduced disease in plants.

Among the abiotic and biotic stress combinations, drought-pathogen and heat-pathogen are the most studied stress combinations. In the following sections, physiological and molecular response of plants to these stress combinations are discussed.

## Physiological response of plants to combined drought and pathogen stress

Interaction of drought and pathogens is mainly influenced by changes in the water potential of plants (Mattson and Haack, 1987; Boyer, 1995). Altered water potential by one stress can increase either the susceptibility or tolerance of plants to the subsequent stress. Drought induced reduction in plant water potential has negative effect on plant interaction with root pathogens. For example, drought led to reduced plant water status in *Phaseolus vulgaris* resulting in more susceptibility of plants to *Macrophomina phaseolina* (causal agent of charcoal rot disease in common bean, Mayek-Perez et al., 2002). It has been shown that *Nicotiana benthamiana* plants challenged with *Sclerotinia sclerotiorum* (causal agent of white mold in beans), exhibit severe cell death, whereas in the drought acclimated plants the extent of cell death was much reduced (Ramegowda et al., 2013). Evidences show the accumulation of abscisic acid (ABA) under combined drought and pathogen stress. For example, drought-stressed *S. lycopersicum* plants that exhibited enhanced resistance against *B. cinerea* also showed the accumulation of ABA (Achuo et al., 2006).

Pathogens can also lower the water potential of plant influencing its responses to drought stress. For example, *Xylella fastidiosa* (causal agent of Pierce's disease in grapes) causes pathogen-induced drought in *Vitis vinifera* by reducing water potential (Choi et al., 2013). One major defense response common to drought and pathogen infection is stomatal closure. Therefore, drought and pathogen-induced stomatal closure can have positive effect on plants under combined drought and pathogen infection (Sawinski et al., 2013). Similarly, drought tolerance of *A. thaliana* plants infected with vascular pathogen *V. longisporum* increased due to increased *de novo* xylem formation resulting in increased water flow (Reusche et al., 2012). The interactive effects of drought and pathogen on plants are discussed in detail by Ramegowda and Senthil-kumar (2015) and Pandey et al. (2015). The effect of concurrent drought on plant pathogen interaction has been discussed in detail by Pandey et al. (2015). Also, Ramegowda and Senthil-kumar (2015) have reviewed the tailored molecular strategies adopted by plants to deal with the stress combination.

## Physiological response of plants to combined heat and pathogen stress

Similar to drought, heat stress can also lead to resistance or susceptibility of plants to pathogen depending on the stress intensity and duration. Heat stress facilitates pathogen spread and cause susceptibility to the diseases (Bale et al., 2002; Luck et al., 2011; Madgwick et al., 2011; Nicol et al., 2011). In wheat, higher mean temperatures observed over a 6 year experimental period correlated with heightened susceptibility to the fungus *Cochliobolus sativus* (causal agent of root rot in wheat, Sharma et al., 2007). In *N. tabacum* and *A. thaliana*, hypersensitive response (HR)—and resistance (*R*)—gene mediated defense responses to *P. syringae* pathovars (causal agent of brown spot in thale cress) and viral elicitors were compromised at high temperatures, allowing increased growth of these pathogens (Wang et al., 2009). Non-acclimation to heat stress causes more susceptibility of plants to pathogen. For example, ornamental plant roots directly exposed to 45°C soil temperatures increased severity of *Phytophthora infestans* (causal agent of root rot in ornamentals, MacDonald, 1991). Heat stress also imparts pathogen resistance. For example, *Cucumis sativus* seedlings exposed to brief heat shock of 50°C resulted in increased resistance to the fungal pathogen *Cladosporium cucumerinum* (causal agent of scab in cucumber; Stermer and Hammerschmidt, 1987). Temperature-dependent suppression of host resistance has been reported for *Tobacco mosaic virus* (TMV; causal agent of mosaic disease in tobacco) and *Tomato spotted wilt virus* (TSWV; causal agent of spotted wilt in tomato). TMV is able to overcome the *N*-gene mediated resistance at temperatures above 28°C in *N. tabacum* (Király et al., 2008), while TSWV is able to suppress TSW, a dominant gene-mediated resistance in *Capsicum chinense* plants at high temperatures (Moury et al., 1998). Thus, heat stress generally leads to suppression of host defense responses along with the other metabolic processes, thereby increasing their susceptibility to pathogens.

## Molecular responses of plants under combined drought and pathogen stress

The transcriptome analysis of *A. thaliana* plants exposed to individual drought, *Turnip mosaic virus* (TuMV, causal agent of mosaic disease in crucifers) and combined drought and TuMV indicated the presence of both shared and unique molecular response in combined stressed plants (Prasch and Sonnewald, 2013). A total of 98 and 157 genes were unique to virus and drought stress whereas 776 genes were unique to combined stress indicating a major reprogramming of plants' defense response under combined stress. Only six genes were common in individual virus and drought stress modulated transcriptome. A total of 160 and 323 genes were commonly regulated under combined stress–drought and combined stress–virus treatment. Totally 112 genes were commonly modulated under all the three stress conditions (Figure 2A). Majority of the commonly regulated genes under combined drought–virus treatment and individual stresses were metabolism related genes (Supplementary Figure 4A). Functional categorization of these commonly regulated genes on the basis of protein classes revealed that the shared response was dominated by protein class constituting oxidoreductases and membrane transporters (Supplementary Figure 4B).

Down-regulation of photosynthetic genes and up-regulation of stress responsive genes constituted the major shared molecular response between individual and combined stressed plants. The combined stress treatment led to up-regulation of 72 stress specific genes as compared to 16 and 29 genes specifically up-regulated under individual drought and virus infection. Virus infection and combined stress treatment lead to up-regulation of *PR* genes. However, *PR* genes were down-regulated under individual drought treatment (Prasch and Sonnewald, 2013). The overview of the expression changes related to metabolic pathways in *A. thaliana* plants during the combined drought and virus infection using MapMan software (Supplementary Figure 5) revealed the up regulation of genes involved in carbohydrate and lipid metabolism. The genes involved in flavonoid metabolism were found to be strongly up-regulated (Prasch and Sonnewald, 2013).

The analysis of *Vitis vinifera* plants subjected to individual drought stress, *X. fastidiosa* infection and combined drought and *X. fastidiosa* infection also revealed down regulation of transcripts involved in photosynthesis, nutrient assimilation, and cellular homeostasis (Choi et al., 2013). The transcriptome analysis of plants in this case largely reflects the exacerbation of disease development by drought stress. Transcript analysis of individual and combined stress treated plants showed a time dependent transcriptional modulation. Whereas early response did not show major changes in the transcriptome, increased stress exposure led to modulation of nearly 700 genes. *X. fastidiosa* infection and drought stress led to some common changes in transcriptome which included up-regulation of ABA and JA synthesis-, pathogenesis related-, and phenylpropanoid and flavonoid biosynthesis-related genes (Choi et al., 2013).

The genes specifically up-regulated by bacterial infection included the ones encoding PR proteins, chitinases, thaumatin like proteins, and lipid-transfer proteins. A characteristic response to *X. fastidiosa* infection was the up-regulation of aquaporin gene which was not observed under drought only treatment. The bacterial infection also led to up-regulation of gene encoding galactinol synthase (GOLS) which is responsible for synthesis of osmoprotectants galactinol and raffinose. The response characteristically seen under combined stresses in *V. vinifera* plants consisted of early induction of ABA biosynthesis gene, 9-cis epoxycarotenoid dioxygenase (*NCED*), and calceneurin B like interacting protein kinase (*CIPK*). Overall, drought stress and bacterial infection in this case led to activation of ABA mediated drought response that led to enhanced development of disease (Choi et al., 2013).

Thus, in both the above instances, transcriptome of plants challenged with combination of drought and viral infection was more affected by the pathogen signifying the dominant effect of biotic over drought stress. In both cases, abiotic stress enhanced the susceptibility of plants to pathogen infection. In the latter case, however, pathogen produced effects similar to drought stress as was reflected by up-regulation of ABA related genes and accumulation of osmoprotectants under individual bacterial treatment. In both instances, transcripts specifically modulated under combined stress treatment outnumbered the drought and pathogen specific as well as commonly regulated transcripts between the two stress conditions. This clearly shows that combined stress is perceived by plants as a new stress leading to major redirection of gene expression in the combined stressed plants.

## Molecular responses of plants under combined heat and pathogen stress

Although physiological effects of combined heat and pathogen on plants has been studied in a number of cases (Bale et al., 2002; Sharma et al., 2007; Wang et al., 2009; Luck et al., 2011; Madgwick et al., 2011; Nicol et al., 2011), molecular response of plants exposed to combined heat and pathogen has been discussed only in a study by Prasch and Sonnewald (2013). In coherence with earlier reports (MacDonald, 1991; Wang et al., 2009), Prasch and Sonnewald (2013) reported that *A. thaliana* plants subjected to combination of heat and *Turnip mosaic virus* (TuMV) infection were more susceptible to viral infection. The authors observed that combination of heat and viral infection led to enhanced transcript accumulation of P3 gene, which is a marker for viral replication (Kim et al., 2010) suggesting more viral replication in combined stressed plants. Microarray analysis of individually and combined stressed *A. thaliana* plants revealed the presence of 190, 920, and 823 unique genes in the transcriptome of heat alone, virus alone, and combined stress treated plants, respectively. Out of the total modulated genes, 88 were commonly regulated under combined stressed and individual virus infected plants and 46 transcripts were common in combined stressed and individual heat stressed plants. The number of transcripts common between heat and combined stressed plants was far higher and was estimated to be 2340. This shows that molecular response of combined stressed plants was majorly governed by heat stress (Figure 2B). A total of 215 transcripts were commonly modulated under all the three stress conditions. Functional classification of transcripts commonly modulated under virus alone and combined stress treated plants revealed that the majority of commonly modulated genes belonged to class of metabolism related genes (Supplementary Figure 6). The virus alone, heat alone and combined heat and virus infection led to up-regulation of 29, 110, and 108 stress responsive transcripts, respectively (Prasch and Sonnewald, 2013). The individual and combined stressed plants shared molecular responses like down-regulation of photosynthetic genes, and differential expression of toll/interleukin1 receptor-nucleotide binding site and leucine rich repeat (TIR-NBS-LRR) genes (Prasch and Sonnewald, 2013).

The individually and combined stressed plants also showed some contrasting molecular responses. For example, virus infection led to up-regulation of *PR1, PR2, PR5*, whereas these genes were down-regulated under combined heat and virus infection further substantiating the heat mediated suppression of basal defense mechanism. The virus alone treatment also led to up-regulation of cell wall bound invertases. However, under heat and combined heat and virus infection, the expression of cell wall bound invertases was down-regulated and that of vacuolar and cytosolic invertases was up-regulated pointing toward the intracellular hydrolysis of sugars in heat stressed and combined stressed plants (Prasch and Sonnewald, 2013). The metabolic overview map generated through MapMan (Supplementary Figure 7) revealed the slight down regulation of genes involved in carbohydrate and lipid metabolism, photosynthesis and mitochondrial electron transport under combined heat and virus infection in *A. thaliana* plants (Prasch and Sonnewald, 2013).

## Cross talk between abiotic and biotic stress defense response and its extrapolation to combined stress response

**Figure 3:**
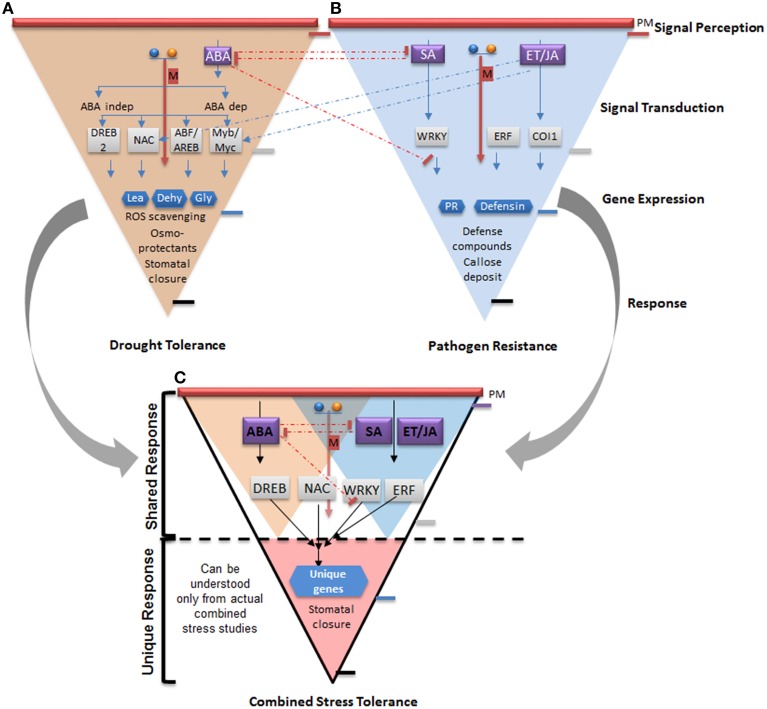
**Model representing cross talk between drought and pathogen stress signaling pathway from individual stress studies and its relation to shared and unique response pathway provoked under combined stress**. The two inverted triangles in the upper panel represents the drought stress **(A)** and biotic stress signaling pathway **(B)** consisting of signal perception, signal transduction, gene expression and response generation steps with the representative gene products. The inverted shape represents the general response (wide upper side) at the stress perception and signal transduction stage culminating to a specific response (tapering lower end) contributing to tolerance to a particular stress. The response of the plants to combined drought and pathogen stress consist of both the shared (responses common to drought and pathogen stress) and unique responses as represented by the triangle **(C)**. The overall response of plants to the combined stress is governed by the key players involving ROS, Ca^2+^, MAPKs, and the different transcription factors as well as some unique genes regulating the tailored responses. The shared responses can be deciphered by understanding the cross talk between the drought and the pathogen stress tolerance signaling networks whereas dedicated studies are required to understand the unique responses. The yellow colored small triangle represents the responses shared between drought and combined stress whereas the blue triangle represents the responses shared between pathogen and combined stress. The overlapping area between the two small triangles represents the responses shared by the drought and pathogen stress. The red colored triangle at the apex represents the unique response under the combined stress. The blue and yellow color spheres represent the calcium and ROS molecules. The red color arrow represents the MAPK pathway. The dashed arrows indicate the cross talk wherein the red colored dashed arrows represent the suppression and blue dashed arrows represent the activation of the respective stress responsive genes. PM, plasma membrane; ABA, abscisic acid; SA, salicylic acid; JA, jasmonic acid; ET, ethylene; M, MAPK pathway; ABA dep, ABA dependent pathway; ABA indep, ABA independent pathway; DREB, dehydration responsive element binding; NAC, NAM-ATAF and CUC 6 transcription factor; ABF, ABA binding factor; AREB, ABA responsive element binding; Myb, myeloblastosis; Myc, myelocytomatosis; ERF, ethylene responsive factor; WRKY stands for the first four amino acids (tryptophan [W], arginine [R], lysine [K], and tyrosine [Y] of the heptapeptide WRKYGQK, which is the hall mark of WRKY proteins; COI1, coronatine insensitive 1; LEA, late embryogenesis; Gly, glyoxylase; dehyd, Dehydrin; PR, pathogenesis related; ROS, reactive oxygen species.

Apart from the unique gene expression mediated by different stress conditions, there can be various points of cross talk between the stress signaling pathways (Figures 3A,B). As defined by Knight and Knight (2001) cross talk refers to “any instance of two signaling pathways from different stressors that converge.” The signaling pathways for abiotic and biotic stresses share common elements including ROS (Møller et al., 2007; Wong and Shimamoto, 2009), calcium ions (Galon et al., 2010), transcription factors (Walley and Dehesh, 2010), hormones (Fonseca et al., 2009; Ton et al., 2009), and mitogen-activated protein kinase (MAPK) cascades (Pitzschke et al., 2009). Identification of cross talk between signaling pathways has been crucial in envisaging and strengthening our understanding on regulation of plants response to a particular stress condition. In recent years, the studies dealing with cross talk between abiotic and biotic stress signaling pathways have shed light on genes or gene products that are involved in two different stress conditions and thus are a part of shared response. The transgenic overexpression or down-regulation of these genes showed that they play crucial role in conferring tolerance to more than one abiotic or biotic stress conditions (Supplementary Table 2). Thus, these genes can be significant in providing resistance to plants against combined biotic and abiotic stresses.

Ca^2+^ and ROS are ubiquitous components of both abiotic and biotic stress signaling pathways. Genes involved in ROS and Ca signaling constitutes an important part of the shared molecular response of the combined stress plants (Rizhsky et al., 2004; Johnson et al., 2014). Analysis of different calcium dependent protein kinase (CDPK) genes in *T. aestivum* showed that out of 12 CDPKs which were responsive to *Blumeria graminis* pv. *tritici* (causal agent of powdery mildew in wheat) infection, eight also responded to abiotic stresses substantiating them as an important point of cross talk (Li et al., 2008). Similarly genes involved in ROS scavenging pathway like *APX*, have been shown to impart tolerance against various abiotic and biotic stresses (Sarowar et al., 2005; Choi and Hwang, 2012).

A number of transcription factors belonging to myeloblastosis (MYB) transcription factors family e.g., OsMYB4, ethylene responsive factors (ERF) like GmERF and botrytis-susceptible1 (BOS1) are important regulators of different hormone signaling pathways and have a role in imparting biotic and abiotic stress resistance to plants (Mengiste et al., 2003; Iriti et al., 2007; Zhang et al., 2009). A number of WRKY genes like *O. sativa* WRKY89 (OsWRKY89), *Capsicum annum* WRKY40 (CaWRKY40), MAPK like *Gossypium hirsutum* MPK16 (GhMPK16), and OsNAC6 have been successfully used to impart biotic and abiotic stress resistance to plants (Supplementary Table 2). Similarly MAPK and NAM-ATAF and CUC 6 (NAC) transcription factors also play a crucial role in regulating biotic and abiotic stress response of plants. The phytohormone ABA has also been known to be an important modulator of plants responses to various abiotic and biotic stress conditions (Tuteja, 2007; Ton et al., 2009). The fact that these “cross talk” genes regulate both biotic and abiotic stress response of plants points toward their probable importance in conferring combined stress tolerance to plants. However, this needs to be validated by actual combined stress studies wherein expression of these genes under combined stress needs to be investigated.

The transcriptome analysis of *A. thaliana* plants subjected to combined drought and TuMV infection has revealed the presence of some of the genes involved in cross talk between individual stresses as a part of shared response under combined stress. For example, genes like *AtERF1b, AtERF1a, WRKY38*-related (WRKY transcription factor 38), glutathione-S-transferase F12 (*GSTF12*), mitogen activated protein kinase 9 (*AtMAPKK9*), *MAPKK16* are common to drought and combined virus and drought stress response of *A. thaliana* plants subjected to combined drought and TuMV infection (Prasch and Sonnewald, 2013). Thus, individual stress studies which give an indication about molecules involved in the cross talk can be important to gain insights into the shared response under combined stress treatment (Figure 3C).

## Conclusion and future perspectives

The stress response mechanism of plants against the abiotic and biotic stress combinations is governed by interaction between responses evoked by individual stresses at both physiological and molecular level. As already stated, the interaction is governed by factors like severity of stresses, age of plant, and whether the plant is tolerant or susceptible to any one of the individual stress. Even plants belonging to same genus may show different molecular response to a stress combination (Aprile et al., 2013). If the two stresses under a stress combination lead to same kind of physiological changes in plants, the overall effect of stress combination becomes additive causing enhanced damage to plants. However, stress combinations can have entirely different effects on physiological and molecular processes of plants. The overall response of plants to stress combination is apparently governed by the more severe stress. The interaction between two stresses sometimes leads to a completely unique response that ensures best utilization of plant energy resources. Thus, adaptation mechanism to combined stresses consists of both shared and unique responses. The identification of genes involved in shared and unique response under combined stresses would be an important step toward developing a comprehensive understanding on the mechanism of combined stress tolerance in plants. Further analysis and characterization of these genes would help in choosing the right candidates among shared and unique response genes which can be potential targets for conferring combined stress tolerance to plants.

The increasing demand for food, deteriorating environmental conditions as well as emergence of newly evolved pathogens have necessitated the development of crops which are better equipped to deal with biotic and abiotic stresses and produce better yields. The fact that occurrence of combination of stresses instead of individual stress are important challenges for crop production demands thorough and intensive studies to understand plants response to stress combinations. A couple of studies in this direction throwing light on shared and unique responses of plants under combined stresses have already been published. It is now required to extend these studies to major crop plants. For proper understanding of plants responses to combined stress which occur under field conditions, the experiments should be carefully designed so that they nearly mimic the field conditions. It is also pertinent to identify the stages vulnerable to the combined stresses by studying the stage specific effect of combined stresses on the transcriptome of different plants. The transcriptomic analysis of plants under combined stresses can generate useful and substantial information regarding common and unique genes modulated under combined stresses. The advances in NGS and high throughput sequence analysis platforms for precise detection and accurate quantification of even small changes in the transcriptome as well as the recently developed genetic engineering tools can be useful in exploring the molecular responses of plants under combined stresses.

### Conflict of interest statement

The reviewer Yasuhiro Ishiga declares that, despite having previously collaborated with the authors Muthappa Senthil-Kumar and Venkategowda Ramegowda, the review process was conducted objectively. The reviewer Ramu S. Vemanna also declares that, despite having previously collaborated with the author Muthappa Senthil-Kumar, the review process was conducted objectively. The authors declare that the research was conducted in the absence of any commercial or financial relationships that could be construed as a potential conflict of interest.
