# Supplementary material for: Shared and unique responses of plants to multiple individual stresses and stress combinations: physiological and molecular mechanisms
Source: Front Plant Sci. 2015 Sep 16;6:723. doi: 10.3389/fpls.2015.00723 (PMC4584981; doi:10.3389/fpls.2015.00723)
Supplement: Supplementary file 1 [file Table1.DOCX]

**Supplementary Tables**

**Supplementary Table 1. Morpho-physiological effects of individual and combined abiotic stresses on plants.**

| **S. No** | **Traits and processes** | **Individual Stress** | | **Combined Stress** | **Reference** |
| --- | --- | --- | --- | --- | --- |
|  |  | Heat | Drought | Heat +Drought |  |
|  | Leaf expansion/Size | Increased | Not affected | Increased | Vile et al., 2012 |
|  | Leaf number | Increased | Decreased | Decreased |  |
|  | Leaf dry matter content | Decreased | Increased | Increased |  |
|  | Leaf insertion angle | Increased | Not affected | Increased |  |
|  | Stomatal density | decreased | Increased | Decreased |  |
|  | Stomatal Index | Decreased | Increased | Decreased |  |
|  |  | Decreases | Decreases | Decreased | Simonneau et al., 1993,  Prasad et al., 2006 |
|  | Biomass allocation | Increased in reproductive parts | Increased in vegetative parts & Roots | Decreased in vegetative, increased in roots & reproductive parts | Vile et al., 2012 |
|  | Photosynthesis | Decreased  RubisCo inactivation | Decreased | Decreased  Affects PSII function | Rizhsky et al., 2002, Sainz et al., 2010 |
|  | Respiration | Increased | Decreased | Increased | Rizhsky et al., 2002, |
|  | ABA content | Increased | Increased | Increased | Vile et al., 2012 |
|  |  | Heat | Salinity | Heat +Salinity |  |
|  | Growth (fresh weight of roots and shoots) | Slightly decreased | Decreased | Decreased but lesser extent as compared to salt stress alone | Rivero et al., 2014 |
|  | Na^+^ Uptake (stem and leaves) | No change | Increased in stems and leaves | Increased in stems and leaves but to lesser extent as compared to salt stress alone |  |
|  | Na^+^ Uptake (root) | No change | Increased | Increased more than that observed in salt stress alone |  |
|  | K^+^ uptake in stem, leaves and roots | No change | Decreased | Decreased leaves but to lesser extent as compared to salt stress alone |  |
|  | Water potential | Decreased | Decreased | More than salt and heat |  |
|  | CO_2_ assimilation | Decreased | Decreased | More than salt but less than heat |  |
|  | Transpiration rate | Increased | Decreased | Unaffected |  |
|  | Photosynthetic efficiency (Fv/Fm) | Decreased | Decreased | Decreased but to lesser extent as compared to salt stress alone |  |
|  | Photosynthetic efficiency (PS-II) | Decreased | Decreased | Decreased but to lesser extent as compared to salt stress alone |  |
|  | Osmolyte accumulation | Proline, Glycine betaine | Proline, Choline | Glycine betaine, Sucrose, Starch, Trehalose |  |
|  | Proline metabolism | All enzyme activities decreased | Synthesis of proline from glutamate increased.  Proline degradation decreased | Synthesis of proline from OAT increased.  Proline degradation increased |  |
|  | Glycine betaine synthesis | Increased | Decreased | Increased |  |
|  | Lipid peroxidation | Increased | Increased | Increased |  |
|  |  | Drought | Salinity | Drought + Salinity |  |
|  | Plant growth | Decreased | Decreased | Decreased | Ahmed et al., 2013 |
|  | Photosynthetic efficiency | Decreased | Decreased | Decreased |  |
|  | Chlorophyll content | Chl a, b reduced | Chl a, b reduced | Chlorophyll b reduction |  |
|  | Na^+^ Uptake in root | - | Increased | Increase & more than salt stress alone |  |

Supplementary Table 2: List of genes involved in biotic and abiotic stress crosstalk and the effect of their modulation on abiotic and biotic stress tolerance of plants

| **S. No** | **Plant species** | **Gene name and corresponding Gene IDs** | **Response to abiotic stress in transgenic plants** | **Response to biotic stress in transgenic plants** | **Associated signaling pathway** | **Reference** |
| --- | --- | --- | --- | --- | --- | --- |
|  | *Arabidopsis thaliana* | Vascular plant one-zinc-finger proteins (VOZs)**^#^**  (AT1G28520, AT2G42400) | Cold and drought-stress tolerance increased in *voz1voz2* double mutant. | Resistance to *Colletotrichum higginsianum* (BS1**)*, *Pseudomonas syringae* (BS2*) decreased in *voz1voz2* double mutant as compared to wild type. | C-repeat binding factors/Drought responsive element binding 1 (CBF/DREB1) pathway, Salicylic Acid (SA) signaling | Nakai et al., 2013 |
|  | *Arabidopsis thaliana* | *Capsicum annuum* extracellular peroxidase 2 (CaPO2); (DQ489711) | Overexpression (OE) confers enhanced  tolerance to salinity, drought, oxidative stress | OE confers increased resistance against *Alternaria brassicicola.* (BS1) as compared to wild type. | Abscisic acid (ABA) signaling | Choi and Hwang, 2012 |
|  | *Nicotiana tabacum* | *Capsicum annuum* Ascorbate peroxidase-like 1 gene (CAPOA1); AF442387 | OE confers increased tolerance to methyl viologen-mediated oxidative stress | OE confers enhanced resistance to *Phytophthora nicotianae* (BS3*) as compared to wild type. | Reactive oxygen species (ROS) signaling | Sarowar et al., 2005 |
|  | *Arabidopsis thaliana* | *Capsicum annuum* Antimicrobial peptide 1 (CaAMP1); AY548741 | OE confers enhanced tolerance to high salinity and drought | OE confers enhanced resistance to *Fusarium oxysporum* f. sp. *matthiolae* (BS1), *Pseudomonas syringae* (BS2), *Hyaloperonospora parasitica* (BS3) as compared to wild type. | ABA signaling | Lee and Hwang, 2009;  Lee et al., 2008 |
|  | *Arabidopsis thaliana* | Heat shock factor A 1b (HSFA1b); AT5G16820 | OE confers enhanced tolerance to drought | OE confers enhanced tolerance *Pseudomonas syringae* (BS2) as compared to wild type. | ROS signaling | Bechtold et al., 2013 |
|  | *Arabidopsis thaliana* | *Capsicum annuum* ERF/AP2-type transcription factor (CaPF1); AY246274 | OE confers tolerance against cold stress | OE confers increased resistance to *Pseudomonas syringae* (BS2) as compared to wild type. | Ethylene signaling | Yi et al., 2004 |
|  | *Oryza sativa* | *Zea mays* G box Factor 14-6 (ZmGF14-6)^# #^ ; (S77133.1) | OE confers enhanced tolerance  to drought | OE confers higher susceptibility to infection by *Fusarium verticillioides*, *Magnaporthe oryzae* (BS1) as compared to wild type. | Associated signaling pathway not known | Campo et al., 2011 |
|  | *Arabidopsis thaliana* | Multiprotein bridging factor 1a (MBF1a); AT2G42680 | OE confers enhanced tolerance  to salt stress | OE confers enhanced tolerance *Botrytis cinerea (BS1)* as compared to wild type. | Ethylene /Jsmonic Acid (ET/JA)-response signaling | Kim et al., 2007 |
|  | *Musa spp* | *Musa paradisiaca* WRKY71, (MusaWRKY71);  HM640261 | OE confers enhanced tolerance to oxidative and salt stress | OE confers enhanced tolerance *Fusarium oxysporum* (BS1) as compared to wild type. | SA and ET/JA signaling | Shekhawat and Ganapathi, 2013 |
|  | Nicotiana tabacum | *Capsicum annuum* WRKY40 (CaWRKY40);  AAX20040 | OE confers tolerance to heat shock | OE confers enhanced tolerance *Ralstonia solanacearum* (BS2) as compared to wild type. | SA and ET/JA signaling | Dang et al., 2013 |
|  | *Oryza sativa* | *Oryza sativa* WRKY89 (OsWRKY89);  AY781112 | OE confers enhanced tolerance to UV-B stress | OE confers enhanced tolerance to *Magnaporthe grisea* (BS1) as compared to wild type. | SA and ET/JA signaling | Wang et al., 2007 |
|  | *Nicotiana tabacum* | *Brassica juncea* Annexin (AnnBj1); DQ191753 | OE confers tolerance to dehydration, salt, heavy metal and oxidative stress | OE confers enhanced resistance to *Phytophthora parasitica* (BS3) as compared to wild type. | ROS signaling | Kumar et al., 2008 |
|  | *Oryza sativa* | *Oryza sativa*  NAM, ATAF and CUC 6 (OsNAC6);  AB028185 | OE confers tolerance to dehydration and high-salt stresses | OE confers enhanced resistance to *Magnaporthe grisea* (BS1) as compared to wild type. | ABA signaling | Nakashima et al., 2007 |
|  | *Triticum aestivum* | *Triticum aestivum* Pathogen induced membrane protein1 (TaPIMP1); EF587267 | OE confers enhanced resistance to the drought stress | OE confers enhanced resistance to *Bipolaris sorokiniana* (BS1) as compared to wild type. | ABA- and SA-signaling | Zhang et al., 2012 |
|  | *Nicotiana tabacum* | *Glycine maize* Ethylene responsive factor (GmERF3); EU681278 | OE confers tolerance to high salinity and dehydration stresses | OE enhanced resistance against infection by *Ralstonia solanacearum* (BS2), *Alternaria alternate* (BS1) and *Tobacco mosaic virus* (BS4*) as compared to wild type. | ET signaling | Zhang et al., 2009 |
|  | *Nicotiana tabacum* | *Trichoderma harzianum* Endochitinases *(*CHIT33 and CHIT42); EF439839, S78423 | OE confers tolerance to salinity, and heavy metals | OE confers broad resistance to *Rhizoctonia. solani* (BS1)and *Pseudomonas syringae* pv *tabaci* (BS2) as compared to wild type. | Not known | Dana et al., 2006 |
|  | *Nicotiana tabacum* | *Oryza sativa* Dehydration responsive element Binding 1b (OsDREB1B);  AY785894 | OE imparts freezing and drought tolerance | OE enhanced resistance to *Tobacco streak virus* (BS4) infection as compared to wild type. | ABA signaling | Gutha and Reddy, 2008 |
|  | *Arabidopsis thaliana* | *Capsicum annum* Lipid Transfer Protein 1 (CALTPI); AF208832 | OE imparts salinity and drought tolerance | OE confers resistance against infection by *Pseudomonas syringae pv*. tomato (BS2)and *Botrytis cinerea* (BS1) as compared to wild type. | Lipid signaling | Jung et al., 2005 |
|  | *Arabidopsis thaliana* | *Gossypium hirsutum* Mitogen activated Protein Kinase 16 (GhMPK16);  FJ966896 | OE imparts drought tolerance | OE confers resistance to *Colletotrichum nicotianae*, *Alternaria alternate* (BS1) and *Pseudomonas solanacearum* (BS2) as compared to wild type. | Mitogen activated protein kinase signaling | Shi et al., 2011 |
|  | *Nicotiana tabacum* | *Oryza sativa* RING-H2 finger protein gene (OsBIRF1)  LOC_Os02g50930 | OE imparts oxidative tolerance | OE enhanced disease resistance against *Tobacco mosaic virus* (BS4) and *Pseudomonas syringae* pv. *tabaci* (BS2) as compared to wild type. | SA and JA signaling | Liu et al., 2008 |
|  | *Solanum lycopersicum* | *Solanum lycopersicum* Abscisic acid-induced myb1(SlAIM1); EU934734 | Down regulation imparts increased sensitivity to salt and oxidative stress | Down regulation imparts increased susceptibility to *Botrytis cinerea* (BS1) as compared to wild type. | ABA signaling | Abuqamar et al., 2009 |
|  | *Oryza sativa* | *Oryza sativa Calcium Dependent Protein Kinase*  (OsCPK12)^# #^ ; FB924751 | OE imparts salinity tolerance | OE imparts susceptibility to *Magnaporthe grisea* (BS1) as compared to wild type. | Calcium signaling / ROS signaling | Asano et al., 2012 |
|  | *Solanum lycopersicum* | *Solanum lycopersicum* Stress-related NAC1 (SlSRN1)  Solyc12g056790 | Down regulation imparts increased tolerance against oxidative and drought stresses | Down regulation imparts susceptibility to *Botrytis cinerea* (BS1) and *Pseudomonas syringae* DC3000 (BS2) as compared to wild type. | SA/JA signaling | Liu et al., 2014 |
|  | *Arabidopsis thaliana* | Botrytis-Susceptible1 (BOS1); AT3G06490 | *bos1* mutant exhibit susceptibility to drought, salinity and oxidative stress | *bos1* mutant exhibit susceptibility to *Alternaria brassicicola* (BS1), *Pseudomonas syringae pv* tomato(BS2) and *Peronospora parasitica* (BS3) as compared to wild type. | ROS signaling | Mengiste et al., 2003 |
|  | *Arabidopsis thaliana* | RAV1 (Related to ABI3/VP1); AT1G13260 | OE imparts salinity and drought tolerance | OE imparts increased resistance to *Pseudomonas syringae pv.* tomato DC3000 (BS2) as compared to wild type. | SA signaling | Sohn et al., 2006 |
|  | *Nicotiana tabacum* | *Thaumatococcus daniellii*  Thaumatin; AF3550098 | OE imparts salinity and drought tolerance | OE imparts enhanced resistance against *Pythium aphanidermatum* and *Rhizoctonia solani* (BS1) as compared to wild type. | Not known | Rajam et al., 2007 |
|  | *Arabidopsis thaliana* | *Oryza sativa* WRKY45 (OsWRKY45); GQ331932 | OE imparts enhanced tolerance to salt and drought stresses | OE imparts enhanced resistance to *Pseudomonas syringae* tomato DC3000 (BS2) as compared to wild type. | ABA signaling | Qiu and Yu, 2009 |
|  | *Solanum lycopersicum* | *Oryza sativa* Myeloblastosis 4 (OsMYB4); D88620 | OE imparts enhanced tolerance to drought stresses | OE imparts resistance to *Tomato mosaic virus* (ToMV) (BS4) as compared to wild type. | ABA signaling | Iriti et al., 2007 |

*Refers to the different types of pathogens- BS1 refers to fungal; BS2 refers to bacterial and BS3 refers to oomycete pathogens;

^#^ Negative regulator of abiotic stress tolerance and positive regulator of biotic stress tolerance

^# #^ Positive regulator of abiotic stress tolerance and negative regulator of biotic stress tolerance

**References**

Abuqamar, S., Luo, H., Laluk, K., Mickelbart, M. V., and Mengiste, T. (2009). Crosstalk between biotic and abiotic stress responses in tomato is mediated by the AIM1 transcription factor. *Plant J.* 58, 347–360. doi:10.1111/j.1365-313X.2008.03783.x.

Asano, T., Hayashi, N., Kobayashi, M., Aoki, N., Miyao, A., Mitsuhara, I., and Ichikawa, H. (2012). A rice calcium-dependent protein kinase OsCPK12 oppositely modulates salt-stress tolerance and blast disease resistance. *Plant J.* 69 (1), 26–36. doi:10.1111/j.1365-313X.2011.04766.x.

Bechtold, U., Albihlal, W. S., Lawson, T., Fryer, M. J., Sparrow, P. A. C., Richard, F., Persad, R., Bowden, L., Hickman, R., Martin, C., et al. (2013). Arabidopsis HEAT SHOCK TRANSCRIPTION FACTORA1b overexpression enhances water productivity, resistance to drought , and infection. J. Exp. Bot. 64(11), 3467–3481. doi:10.1093/jxb/ert185.

Campo, S., Messeguer, J., Peris-peris, C., Montesinos, L., Pen, G., and Segundo, B. S. (2011). Expression of the maize ZmGF14-6 gene in rice confers tolerance to drought stress while enhancing susceptibility to pathogen infection.*J. Exp. Bot. 63(2)*, 1–17. doi:10.1093/jxb/err328.

Choi, H. W., and Hwang, B. K. (2012). The pepper extracellular peroxidase CaPO2 is required for salt, drought and oxidative stress tolerance as well as resistance to fungal pathogens. *Planta* 235, 1369–1382. doi:10.1007/s00425-011-1580-z.

Lee, S.C. and Hwang, B. K. (2009). Functional roles of the pepper antimicrobial protein gene , CaAMP1 , in abscisic acid signaling , and salt and drought tolerance in Arabidopsis. *Planta,* 229(2), 383–391. doi:10.1007/s00425-008-0837-7.

Dana, M., Pintor-Toro, J. A., and Cubero, B. (2006). Transgenic Tobacco Plants Overexpressing Chitinases of Fungal Origin Show Enhanced Resistance to Biotic and Abiotic Stress Agents. *Plant Physiol.* 142(2). 142, 722–730. doi:10.1104/pp.106.086140.

Dang, F., Wang, Y., Yu, L. U., Eulgem, T., Lai, Y. A. N., Liu, Z., Wang, X. U., Qiu, A., Zhang, T., Lin, J., et al. (2013). CaWRKY40 , a WRKY protein of pepper , plays an important role in the regulation of tolerance to heat stress and resistance to Ralstonia solanacearum infection ABSTRACT. 757–774. doi:10.1111/pce.12011.

Gutha, L. R., and Reddy, A. R. (2008). Rice DREB1B promoter shows distinct stress-specific responses , and the overexpression of cDNA in tobacco confers improved abiotic and biotic stress tolerance. Plant Mol. Biol. 68 (6), 533–555. doi:10.1007/s11103-008-9391-8.

Iriti, M., Vannini, C., Carravieri, S., Genga, A., Bracale, M., and Faoro, F. (2007). The rice Osmyb4 gene modulates the resistance to tomato mosaic virus in transformed tomato plants improving the quality traits of fruits . ( Abstract / Poster in atti di convegno ). 00, 2–5.

Jung, H. W., Kim, K. D., and Hwang, B. K. (2005). Identification of pathogen-responsive regions in the promoter of a pepper lipid transfer protein gene ( CALTPI ) and the enhanced resistance of the CALTPI transgenic Arabidopsis against pathogen and environmental stresses. Planta 221(3), 361–373. doi:10.1007/s00425-004-1461-9.

Kim, M., Lim, G., Kim, E., Ko, C., Yang, K., Jeong, J., Lee, M., and Soo, C. (2007). Abiotic and biotic stress tolerance in Arabidopsis overexpressing the Multiprotein bridging factor 1a ( MBF1a ) transcriptional coactivator gene. *Biochem Biophys Res Commun.* 354, 440–446. doi:10.1016/j.bbrc.2006.12.212.

Kumar, S., Clark, G. B., Anuradha, S., Handley, C., Roux, S. J., and Bharadwaja, P. (2008). Plant Physiology and Biochemistry Ectopic expression of an annexin from Brassica juncea confers tolerance to abiotic and biotic stress treatments in transgenic tobacco. *Plant  Physiol. Biochem.* 46, 1019–1030. doi:10.1016/j.plaphy.2008.07.006.

Liu, B., Ouyang, Z., Zhang, Y., Li, X., Hong, Y., Huang, L., Liu, S., and Zhang, H. (2014). Tomato NAC Transcription Factor SlSRN1 Positively Regulates Defense Response against Biotic Stress but Negatively Regulates Abiotic Stress Response. *PLoS one* 9. doi:10.1371/journal.pone.0102067.

Liu, H., Zhang, H., Yang, Y., Li, G., Yang, Y., Wang, X., Basnayake, B. M. V. S., Li, D., and Song, F. (2008). Functional analysis reveals pleiotropic effects of rice RING-H2 finger protein gene OsBIRF1 on regulation of growth and defense responses against abiotic and biotic stresses. *Plant Mol. Biol.* 68, 17–30. doi:10.1007/s11103-008-9349-x.

Mengiste, T., Chen, X., Salmeron, J., and Dietrich, R. (2003). The BOTRYTIS SUSCEPTIBLE1 Gene Encodes an R2R3MYB Transcription Factor Protein That Is Required for Biotic and Abiotic Stress Responses in *Arabidopsis*. *Plant Cell* 15, 2551–2565. doi:10.1105/tpc.014167.been.

Nakai, Y., Nakahira, Y., Sumida, H., Takebayashi, K., Nagasawa, Y., and Yamasaki, K. (2013). Vascular plant one-zinc-finger protein 1 / 2 transcription factors regulate abiotic and biotic stress responses in Arabidopsis. *Plant J.* 73 (5), 761–775. doi:10.1111/tpj.12069.

Nakashima, K., Tran, L. P., Nguyen, D. Van, Fujita, M., Maruyama, K., Todaka, D., and Ito, Y. (2007). Functional analysis of a NAC-type transcription factor OsNAC6 involved in abiotic and biotic stress-responsive gene expression in rice. *Plant J*. 51 (4), 617–630. doi:10.1111/j.1365-313X.2007.03168.x.

Qiu, Y., Yu, D. (2009). Over-expression of the stress-induced OsWRKY45 enhances disease resistance and drought tolerance in Arabidopsis. *Environ Exp Bot*. 65, 35–47.

Rajam, M.V., Chandola, N., Goud Singh, D., Kashyap, V., Choudhary, M.L., Sihachakr, D.( 2007). Thaumatin gene confers resistance to fungal pathogens as well as tolerance to abiotic stresses in transgenic tobacco plants. *Biol Plant.* 51, 135-151.

Sarowar, S., Nam, E., Jin, Y., Han, S., Deok, K., Kook, B., and Sheop, J. (2005). Overexpression of a pepper ascorbate peroxidase-like 1 gene in tobacco plants enhances tolerance to oxidative stress and pathogens. *Plant Sci.* 169, 55–63. doi:10.1016/j.plantsci.2005.02.025.

Shekhawat, U. K. S., and Ganapathi, T. R. (2013). MusaWRKY71 Overexpression in Banana Plants Leads to Altered Abiotic and Biotic Stress Responses. *Plos One* 8. doi:10.1371/journal.pone.0075506.

Shi, J., Zhang, L., An, H., Wu, C., and Guo, X. (2011). GhMPK16 , a novel stress-responsive group D MAPK gene from cotton , is involved in disease resistance and drought sensitivity. *BMC Mol. Biol.* 12, 22. doi:10.1186/1471-2199-12-22.

Sohn, H. K., Lee, S. C., Jung, H. W., Hong, J. K., and Hwang, B. K. (2006). Expression and functional roles of the pepper pathogen-induced transcription factor RAV1 in bacterial disease resistance , and drought and salt stress tolerance. *Plant Mol. Biol.* 897–915. doi:10.1007/s11103-006-0057-0.

Wang, H., Hao, Æ. J., Chen, Æ. X., Hao, Æ. Z., Wang, Æ. X., Lou, Y., Peng, Æ. Y., and Guo, Æ. Z. (2007). Overexpression of rice WRKY89 enhances ultraviolet B tolerance and disease resistance in rice plants. *Plant Mol. Biol.* 65 (6), 799–815. doi:10.1007/s11103-007-9244-x.

Yi, S. Y., Kim, J., Joung, Y., Lee, S., Kim, W., and Yu, S. H. (2004). The Pepper Transcription Factor CaPF1 Confers Pathogen and Freezing Tolerance in Arabidopsis *Plant Physiol.* 136(1), 2862–2874. doi:10.1104/pp.104.042903.2862.

Zhang, G., Chen, M., Li, L., Xu, Z., Chen, X., Guo, J., and Ma, Y. (2009). Overexpression of the soybean GmERF3 gene , an AP2 / ERF type transcription factor for increased tolerances to salt , drought , and diseases in transgenic tobacco. *J. Exp Bot.* 60, 3781–3796. doi:10.1093/jxb/erp214.

Zhang, Z., Liu, X., Wang, X., Zhou, M., Zhou, X., Ye, X., and Wei, X. (2012). An R2R3 MYB transcription factor in wheat , TaPIMP1, mediates host resistance to Bipolaris sorokiniana and drought stresses through regulation of defense- and stress-related genes. 1155–1170.

Szklarczyk, D., Franceschini, A., Wyder, S., Forslund, K., Heller, D., Huerta-Cepas, J., Simonovic, M., Roth. A., Santos, A3, Tsafou, K.P., Kuhn, M., Bork, P., Jensen, L. J., von Mering, C. (2015). STRING v10: protein-protein interaction networks, integrated over the tree of life. *Nucleic Acids Res.* D447-52. doi: 10.1093/nar/gku1003.

Mi, H., Muruganujan, A., Thomas, P.D. (2012). PANTHER in 2013: modeling the evolution of gene function, and other gene attributes, in the context of phylogenetic trees. *Nucleic Acids Res.* D377-86. doi: 10.1093/nar/gks1118.

Supplementary Table 3. Different combined stress studies included in the review with the details of the stress imposition procedure.

| Plant | Study done in | Age of plant | Stress treatment | | | Reference |
| --- | --- | --- | --- | --- | --- | --- |
|  |  |  | Heat | Drought | Combined |  |
| *Arabidopsis thaliana* | GH | Vegetative | 38⁰C for 6h | Withholding water till 70-75% RWC^##^ | Combined stress was imposed by subjecting the drought stressed plants to heat shock (38⁰C) for 6h | Rizhsky et al., 2004 |
| *Arabidopsis thaliana(*ten different accessions*)* | GH | Whole plant | 30⁰C after emergence throughout development | Withholding water till 25% FC | Combined stress was imposed by subjecting heat stressed plants (30⁰C) to drought stress | Vile et al., 2012 |
| *Triticum aestivum* | GH | Booting Stage | 36/24⁰C^#^ Day 1  40/32⁰C^#^ Day 2 | Withholding water SWC^###^- 12.5% | Combined stress was imposed by subjecting the drought stressed plants to increased temperatures (up to 40°C) | Aprile et al., 2013 |
| *Sorghum bicolor* | GH | Vegetative | 50⁰C for 3h | Witholding water for 4 days | Combined stress was imposed by subjecting the drought stressed plants to 50⁰C for 3h | Johnson et al., 2014 |
| *Hordeum vulgare* (genotypes Arta and Keel) | GH | Reproducti-ve stage | 36⁰C for 1 week | Withholding water SWC- 15% | Combined stress was imposed by subjecting the drought stressed plants to 36 ⁰C for 1 week | Rollins et al., 2013 |

^#^ Day/night

^##^RWC- relative water content

^###^SWC –Soil water content

^####^FC- Field Capacity

GH- Greenhouse
