# Supplementary material for: Shared and unique responses of plants to multiple individual stresses and stress combinations: physiological and molecular mechanisms
Source: Front Plant Sci. 2015 Sep 16;6:723. doi: 10.3389/fpls.2015.00723 (PMC4584981; doi:10.3389/fpls.2015.00723)
Supplement: Supplementary file 2 [file Presentation1.PPTX]

## Slide 1
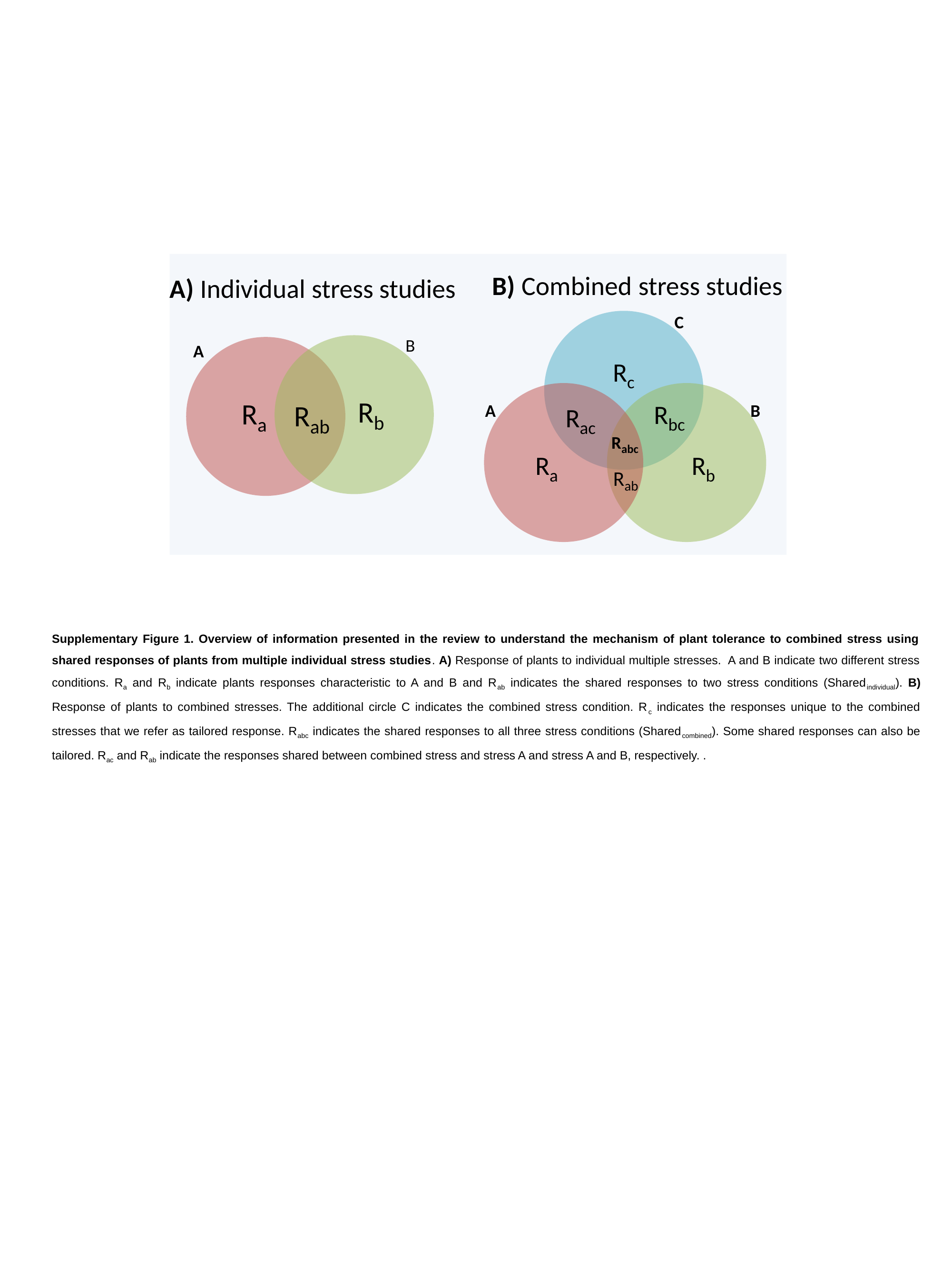

Supplementary Figure 1. Overview of information presented in the review to understand the mechanism of plant tolerance to combined stress using shared responses of plants from multiple individual stress studies. A) Response of plants to individual multiple stresses. A and B indicate two different stress conditions. Ra and Rb indicate plants responses characteristic to A and B and Rab indicates the shared responses to two stress conditions (Sharedindividual). B) Response of plants to combined stresses. The additional circle C indicates the combined stress condition. Rc indicates the responses unique to the combined stresses that we refer as tailored response. Rabc indicates the shared responses to all three stress conditions (Sharedcombined). Some shared responses can also be tailored. Rac and Rab indicate the responses shared between combined stress and stress A and stress A and B, respectively. .
