# Supplementary material for: Shared and unique responses of plants to multiple individual stresses and stress combinations: physiological and molecular mechanisms
Source: Front Plant Sci. 2015 Sep 16;6:723. doi: 10.3389/fpls.2015.00723 (PMC4584981; doi:10.3389/fpls.2015.00723)
Supplement: Supplementary file 3 [file Presentation2.PPTX]

## Slide 1
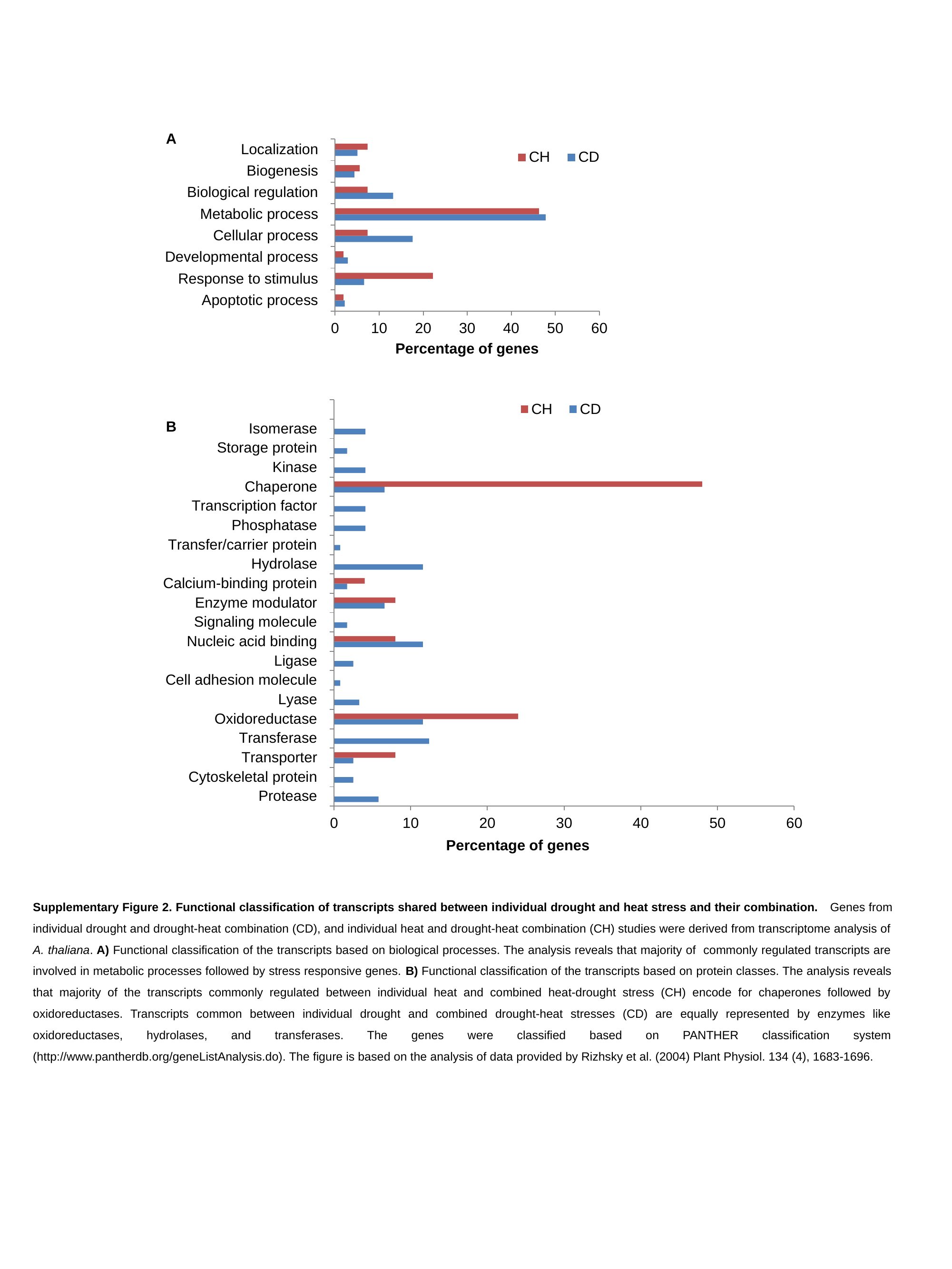

Supplementary Figure 2. Functional classification of transcripts shared between individual drought and heat stress and their combination. Genes from individual drought and drought-heat combination (CD), and individual heat and drought-heat combination (CH) studies were derived from transcriptome analysis of A. thaliana. A) Functional classification of the transcripts based on biological processes. The analysis reveals that majority of commonly regulated transcripts are involved in metabolic processes followed by stress responsive genes. B) Functional classification of the transcripts based on protein classes. The analysis reveals that majority of the transcripts commonly regulated between individual heat and combined heat-drought stress (CH) encode for chaperones followed by oxidoreductases. Transcripts common between individual drought and combined drought-heat stresses (CD) are equally represented by enzymes like oxidoreductases, hydrolases, and transferases. The genes were classified based on PANTHER classification system (http://www.pantherdb.org/geneListAnalysis.do). The figure is based on the analysis of data provided by Rizhsky et al. (2004) Plant Physiol. 134 (4), 1683-1696.
