# Supplementary material for: Shared and unique responses of plants to multiple individual stresses and stress combinations: physiological and molecular mechanisms
Source: Front Plant Sci. 2015 Sep 16;6:723. doi: 10.3389/fpls.2015.00723 (PMC4584981; doi:10.3389/fpls.2015.00723)
Supplement: Supplementary file 5 [file Presentation4.PPTX]

## Slide 1
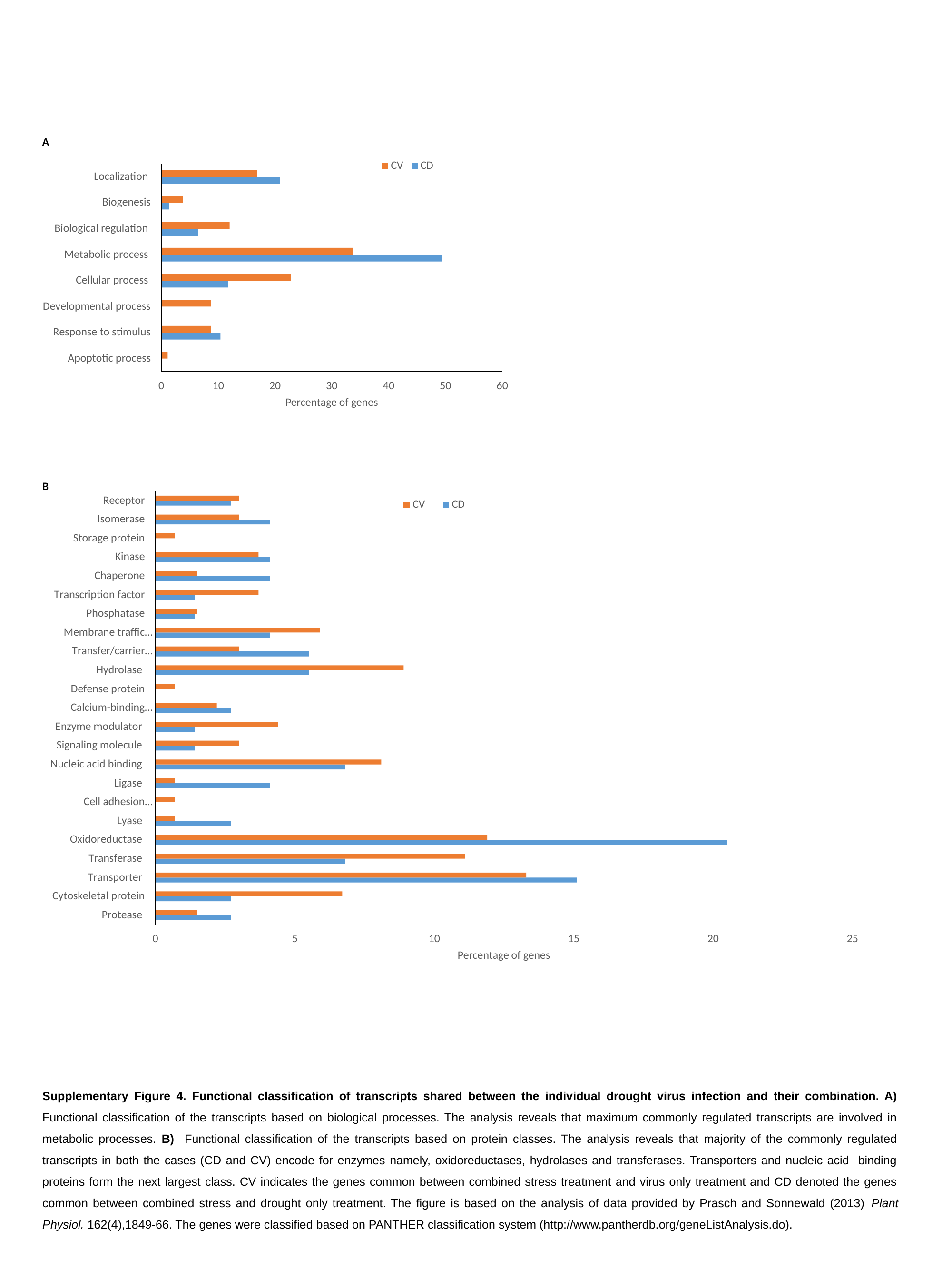

Supplementary Figure 4. Functional classification of transcripts shared between the individual drought virus infection and their combination. A) Functional classification of the transcripts based on biological processes. The analysis reveals that maximum commonly regulated transcripts are involved in metabolic processes. B) Functional classification of the transcripts based on protein classes. The analysis reveals that majority of the commonly regulated transcripts in both the cases (CD and CV) encode for enzymes namely, oxidoreductases, hydrolases and transferases. Transporters and nucleic acid binding proteins form the next largest class. CV indicates the genes common between combined stress treatment and virus only treatment and CD denoted the genes common between combined stress and drought only treatment. The figure is based on the analysis of data provided by Prasch and Sonnewald (2013) Plant Physiol. 162(4),1849-66. The genes were classified based on PANTHER classification system (http://www.pantherdb.org/geneListAnalysis.do).
