# Supplementary material for: Shared and unique responses of plants to multiple individual stresses and stress combinations: physiological and molecular mechanisms
Source: Front Plant Sci. 2015 Sep 16;6:723. doi: 10.3389/fpls.2015.00723 (PMC4584981; doi:10.3389/fpls.2015.00723)
Supplement: Supplementary file 6 [file Presentation5.PPTX]

## Slide 1
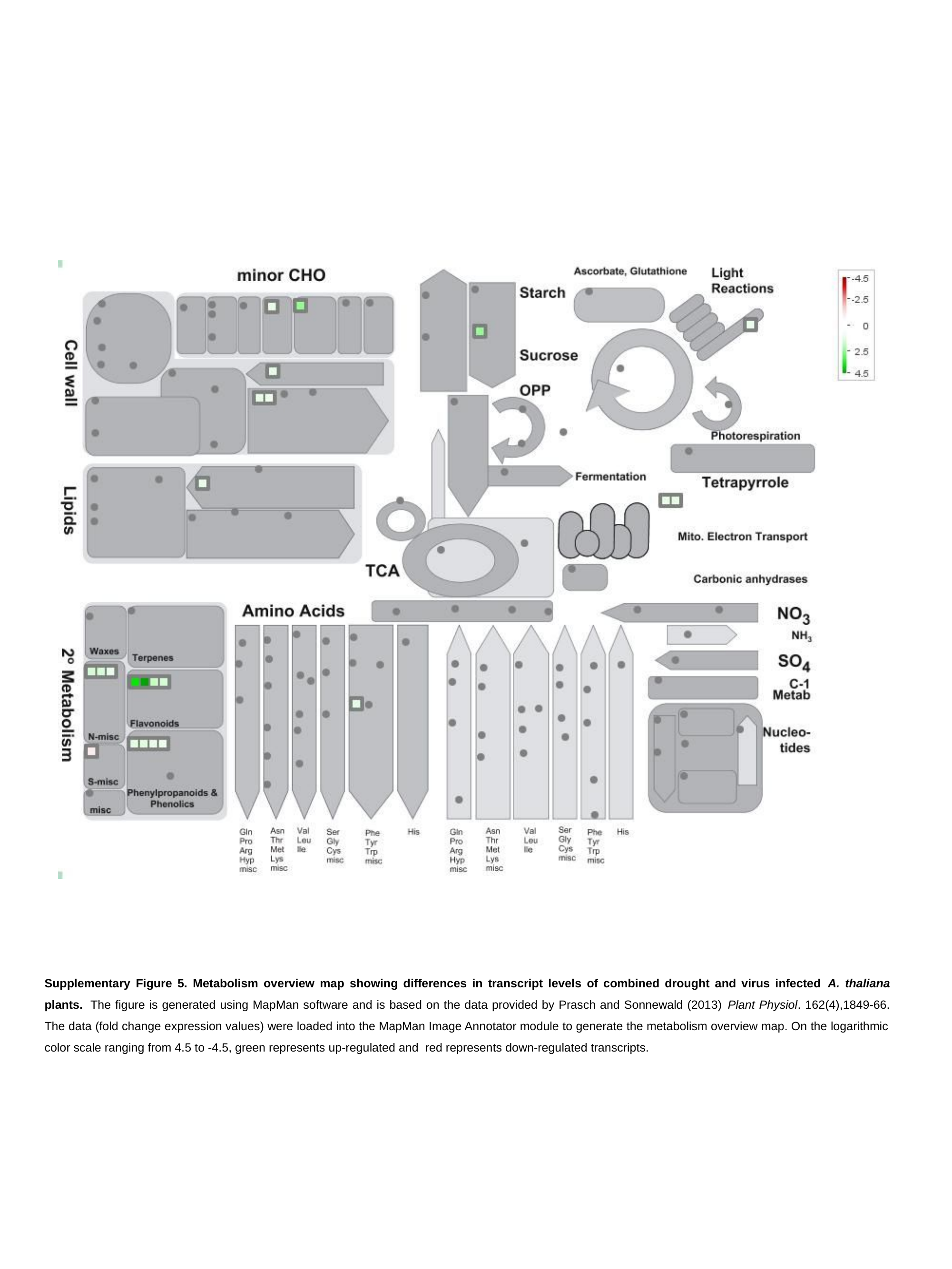

Supplementary Figure 5. Metabolism overview map showing differences in transcript levels of combined drought and virus infected A. thaliana plants.  The figure is generated using MapMan software and is based on the data provided by Prasch and Sonnewald (2013) Plant Physiol. 162(4),1849-66. The data (fold change expression values) were loaded into the MapMan Image Annotator module to generate the metabolism overview map. On the logarithmic color scale ranging from 4.5 to -4.5, green represents up-regulated and red represents down-regulated transcripts.
