# Supplementary material for: Shared and unique responses of plants to multiple individual stresses and stress combinations: physiological and molecular mechanisms
Source: Front Plant Sci. 2015 Sep 16;6:723. doi: 10.3389/fpls.2015.00723 (PMC4584981; doi:10.3389/fpls.2015.00723)
Supplement: Supplementary file 7 [file Presentation6.PPTX]

## Slide 1
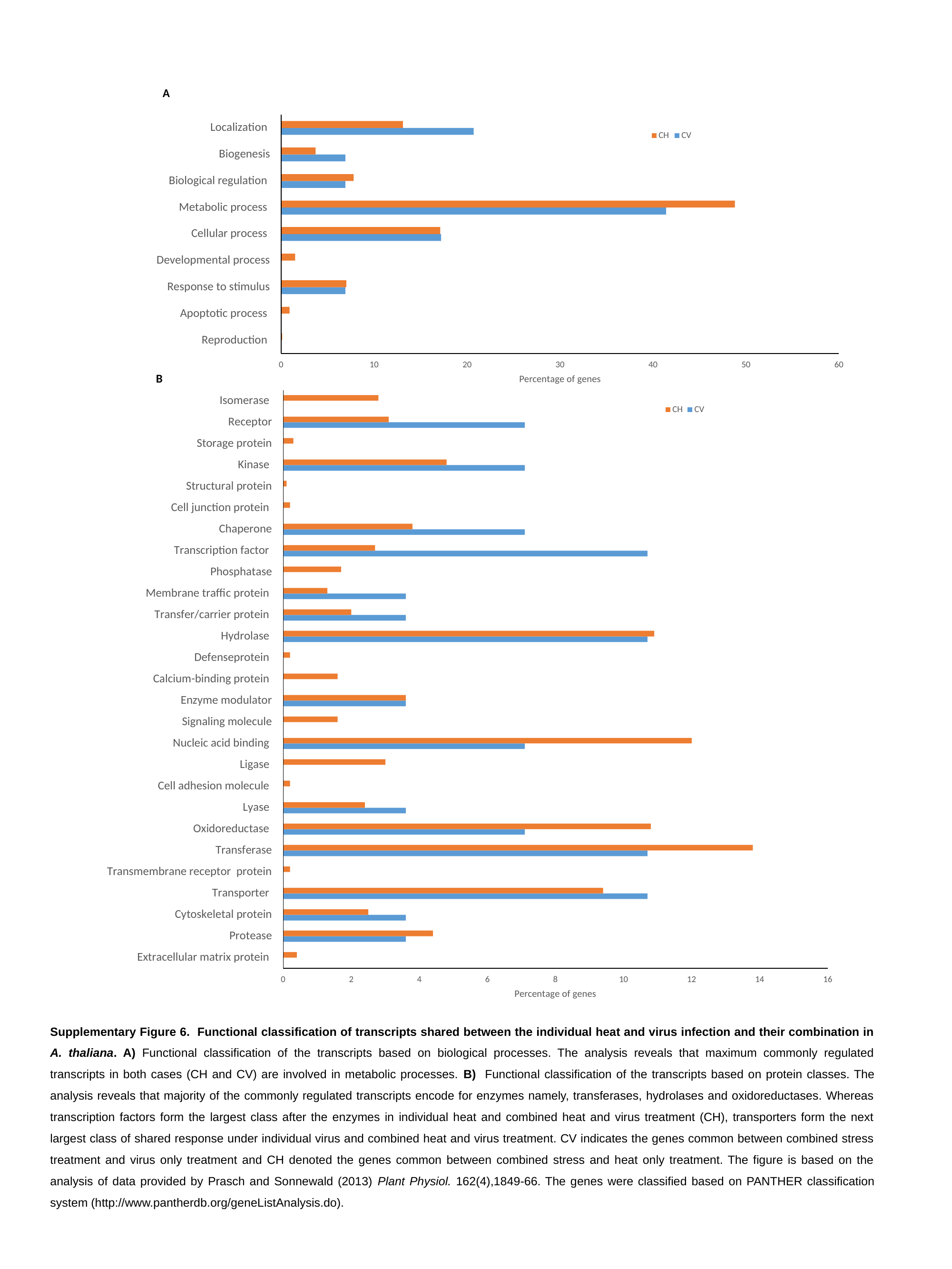

Supplementary Figure 6. Functional classification of transcripts shared between the individual heat and virus infection and their combination in A. thaliana. A) Functional classification of the transcripts based on biological processes. The analysis reveals that maximum commonly regulated transcripts in both cases (CH and CV) are involved in metabolic processes. B) Functional classification of the transcripts based on protein classes. The analysis reveals that majority of the commonly regulated transcripts encode for enzymes namely, transferases, hydrolases and oxidoreductases. Whereas transcription factors form the largest class after the enzymes in individual heat and combined heat and virus treatment (CH), transporters form the next largest class of shared response under individual virus and combined heat and virus treatment. CV indicates the genes common between combined stress treatment and virus only treatment and CH denoted the genes common between combined stress and heat only treatment. The figure is based on the analysis of data provided by Prasch and Sonnewald (2013) Plant Physiol. 162(4),1849-66. The genes were classified based on PANTHER classification system (http://www.pantherdb.org/geneListAnalysis.do).
